# Intergenerational Effects of Neonicotinoid Thiacloprid in Murine Prostate Tissue Are Associated with Epigenetic Alterations in Homeobox Hox Genes

**DOI:** 10.3390/ijms27072921

**Published:** 2026-03-24

**Authors:** Ouzna Dali, Shereen Cynthia D’Cruz, Chaima Diba Lahmidi, Tayeb Mohammed Belkhir, Theo De Gestas, Christine Kervarrec, Pierre-Yves Kernanec, Fatima Smagulova

**Affiliations:** 1 Université de Rennes, EHESP, Inserm, Irset (Institut de Recherche en Santé, Environnement et Travail), UMR_S 1085, F-35000 Rennes, France; daliouzna@gmail.com (O.D.); shereencynthia@gmail.com (S.C.D.); c.diba@protonmail.com (C.D.L.); belkhir.tayeb19@gmail.com (T.M.B.); theodeges@gmail.com (T.D.G.); christine.kervarrec@inserm.fr (C.K.); pierre-yves.kernanec@univ-rennes1.fr (P.-Y.K.); 2 Université de Rennes, OSS, Inserm, UMR_S 1242, 35000 Rennes, France

**Keywords:** neonicotinoids, prostate, epigenetic modifications, *Hox* genes, intergenerational inheritance

## Abstract

Neonicotinoids are widely used pesticides that have caused a catastrophic decrease in bee and bumblebee populations worldwide. In addition to insects, neonicotinoids induce toxic effects in other species, including lizards, birds, and mammals. Previous studies have shown that gestational exposure to thiacloprid promotes transgenerational effects in the testes and thyroid. In this project, we described the epigenetic effects of thiacloprid on prostate tissue in directly exposed F1 and non-directly exposed F3 outbred Swiss male mice. We used paraffin sections for morphological analysis and frozen tissue for immunofluorescence analysis, RT–qPCR, and protein analysis. We purified histones and analyzed them through Western blot. We used ChIP–qPCR for histone H3K4me3 occupancy analysis. A tendency to increase in epithelial hyperplasia in F1 but not in F3 prostate was detected. Elevated levels of phosphorylated histone H3 at serine 10, a marker of mitosis, in both the F1 and F3 prostates were noted. A significant increase in the level of the Ki-67 marker of proliferation was detected in the F1 but not in the F3 anterior prostate. *Hox* gene expression was upregulated in the F1 and downregulated in the F3 prostate. The changes in gene expression were positively associated with histone H3K4me3 alterations at the promoters of the *Hoxa* and *Hoxb13* genes. We determined that regions of *Hox* genes that play important roles in prostate development had altered DNA methylation in the sperm of F1 and F3. These alterations in DNA methylation were negatively related to gene expression. This is an observational study, as it was part of our previous research on the effects of thiacloprid on the testis and thyroid. Our analysis revealed that gestational exposure to thiacloprid induced an increase in cell proliferation in the prostates of directly exposed F1. Some persistent epigenetic alterations in the prostate of F3 males were not associated with phenotypic changes.

## 1. Introduction

Neonicotinoids are widely used pesticides that are intended to control pest populations. The intensive use of neonicotinoids has led to a dramatic decline in bee and bumblebee populations [1]. Neonicotinoids have toxic effects on other species, including lizards, birds, and mammals. In mammals, neonicotinoids are toxic to the central nervous system; specifically, they can bind to acetylcholine receptors [2]. Neonicotinoids have been detected in the environment in countries where they are in use. It has been found that thiacloprid concentrations in surface waters can reach up to ~63.8 ng/L in rainfall water and ~39.7 ng/L in river water in China [3]. In Europe, neonicotinoids were banned in 2018. However, thiacloprid was detected in the Danube River in Europe in aquatic invertebrate species in the range of 0.1–0.39 ppb wet weight in organisms in 2021 [4,5]. Because bees are exposed to thiacloprid via pollen, where the maximum dose could reach as high as 1.48 mg/kg [6], honey has also been found to be contaminated. For example, the presence of thiacloprid was identified in 75% of honey samples from Europe in 2021 [7]. Another study showed that thiacloprid concentration in honey could reach 0.337 mg/kg per sample [8]. Birds are also contaminated by neonicotinoids. A study performed in 2019 demonstrated that in Europe, feathers of house sparrows, *Passer domesticus*, were contaminated by neonicotinoids, with the maximum dose for thiacloprid found to be equal to 8.43 µg/L [9]. Thiacloprid’s presence has also been detected in domestic animals. In dogs’ urine, thiacloprid can reach ~3–10 µg/L and in cats’ < 5 µg/L [10]. Thiacloprid was detected in humans, as well. Human biomonitoring of urinary neonicotinoids and metabolites performed in 225 human samples from China revealed the presence of thiacloprid in the range of 0.038 ± 0.049 ng per mL [11]. In that study, the authors also found that thiacloprid shows the most significant associations with oxidative stress biomarkers [11]. Notably, urinary thiacloprid concentrations differed by sex; women had a higher level of pesticide in their urine [11]. In another study of urine samples from 386 adolescents from China, a geometric mean for thiacloprid was determined as 2.25 µg/g creatinine [12]. Although thiacloprid was banned in 2018 in Europe, a study showed that thiacloprid was found in the hair of small mammals in 2022 [13], and it was detected in farmland birds such as gray partridges and Montagu’s harrier chicks [14] in 2023, suggesting that there is still a persistence of neonicotinoids in nature.

We and other laboratories have shown that neonicotinoid thiacloprid is toxic to the murine thyroid gland; it induces alterations in thyroid gland morphology and interferes with the production of thyroid hormones [15,16]. We showed that gestational exposure to thiacloprid induces transgenerational alterations in the male reproductive system [17]. These effects are mediated by changes in DNA methylation at regions known as super-enhancers and at germ-cell-responsive reprogramming genes (GRRs). GRR genes are essential for the establishment of the germ cell population during embryonic development [18]. Notably, exposure to thiacloprid reduces the level of testosterone in the blood serum of F3 males [18].

This study continues our previous research; in the current study, we focused on the male accessory gland, the prostate. Previously, neonicotinoids were shown to affect prostate tissue. Specifically, it has been determined that the neonicotinoid imidacloprid (IMI) is toxic to human prostate epithelial cells and induces apoptosis and oxidative stress [19]. In an in vivo study, IMI exposure affected the weight of the prostates and led to a decrease in testosterone levels [20]. The Environmental Protection Agency in the USA (EPA) reported that increased prostate weights and prostatic hypertrophy were induced in dogs after 90 days of treatment with thiacloprid. The EPA classified thiacloprid as “likely to be carcinogenic to humans” based on increased uterine tumors in rats, thyroid follicular adenomas in rats, and ovarian tumors in mice (https://www3.epa.gov/pesticides/chem_search/reg_actions/registration/fs_PC-014019_26-Sep-03.pdf accessed on 26 September 2025). This study aims to assess whether thiacloprid could impose negative effects on the prostate. Considering the reduction in testosterone observed in a previous study, we hypothesize that the prostate could be affected.

Many transcription factors, including a family of homeobox *Hox* genes, play important roles in prostate development. *Hoxa10*, *Hoxa13*, *Hoxb13*, and *Hoxd13* have been shown to contribute to the development and regulation of prostate morphogenesis [21,22,23,24]. The loss of *Hoxb13* function resulted in impaired epithelial differentiation in the prostate [23]. Abnormalities in *Hoxa10* functional loss lead to altered prostate morphology [25]. *Nkx3.1* mutations are associated with increased epithelial proliferation in the prostate [26,27]. We suggest that prostate gland processes are regulated by epigenetic mechanisms and therefore perturbation of them during development could have effects on prostate morphology and functioning in adulthood. In addition, several epigenetic chromatin remodeling factors are known to be overexpressed in prostate cancer, such as *Ezh2* [28], *Hdac1* [29,30], and *Kdm5a* [31,32], suggesting their possible role not only in the pathological transformation of the prostate but also in prostate functioning.

In this study, we aimed to reveal the effects of thiacloprid on prostate morphology and to gain insight into the epigenetic mechanisms involved in the regulation of genes important for prostate functioning and development using a mouse model. Although the mouse prostate is anatomically and histologically different from the human prostate [33], there is extensive evidence that the genetic lesions in human prostate cancer can lead to neoplasia in the murine prostate, suggesting that there is a common conserved mechanism of prostate pathology development [33].

Here, we showed that gestational exposure to thiacloprid tended to increase epithelial hyperplasia in the anterior prostate in F1 but not in F3. We detected increased levels of the mitosis marker PHH3 in the prostate of F1 and F3, and the expression of the oncogenesis marker Ki-67 was significantly increased in directly exposed F1 but not in F3 males. The expression of genes encoding transcription factors, hormones, and chromatin factors was altered in both the F1 and F3 anterior prostates. We observed that gene expression and histone H3K4me3 occupancy at promoters of *Hox* and several transcriptional factors were consistently changed in similar directions in exposed mice. Compared with changes in gene expression, alterations in DNA methylation at the promoters of prostate development genes were observed in F1 and F3, mainly in opposite directions. Our study suggests that in gestationally exposed F1 mice, some damaging effects on the murine prostate were observed, but the effects were no longer present in F3 mice despite the persistence of epigenetic marks.

## 2. Results

### 2.1. Experimental Design

The schematic presentation of our study and results is presented in the graphical abstract. In this study, we assessed the multigenerational effects of the neonicotinoid thiacloprid on the prostate. Pregnant outbred Swiss mice were exposed during the embryonic period from E6.5 to E15.5 for a total of 10 days. The thiacloprid dose used in this study was 6 mg/kg body weight/day. The federal agency uses the lowest-observed-adverse-effect level (LOAEL), the lowest concentration of a substance that causes an adverse alteration of morphology or function. The chosen dose was approximately four times lower than the LOAEL established by the EPA for thiacloprid for mice. The LOAEL dose for developmental neurotoxicity of thiacloprid is 25.6 mg/kg/day based on decreased preweaning and postweaning body weights in both sexes and delayed sexual maturation in males (EPA). Mice tolerate higher doses than humans, according to one study [34]; the conversion factor from mouse to human dose is 12.3. Thus, the mouse dose used in our study corresponds to a human dose of 0.49 mg/kg/day. The LOAEL dose of thiacloprid for humans is equal to 2.5 mg/kg/day based on hepatic hypertrophy, cytoplasmic change, thyroid hypertrophy, and retinal degeneration, according to the EPA (Environmental Protection Agency, USA). The studied dose is ~5 times lower than the human equivalent LOAEL dose established by the EPA. In our previous testis studies, we tested a 10 times lower dose than the dose used in the current study [17]. We did not observe effects on testis morphology, spermatozoa number, or testosterone levels. Thus, we tested only the elevated dose of 6 mg/kg/day in the current study. Pregnant outbred Swiss (RjOrl) female mice were treated with *thia* (0 and 6 mg/kg/day) by administering the compound down the esophagus and into the stomach using a gavage needle from E6.5 to E15.5, which corresponds to the somatic-to-germline transition window. Thiacloprid was suspended in olive oil and administered in a volume of 150 µL for each mouse. The control mice were treated with the same volume of oil. The control and *thia*-treated mice are called F0. The progeny of exposed mice is referred to as F1. Both control and exposed F1 generation males were crossed with non-related, untreated females to obtain the F2 generation. Both control and exposed male progeny of F2 were crossed with non-related, untreated females to obtain the F3 generation. For each dose, a minimum of 8 unrelated pregnant F0 female mice were treated, and, for each assay, 4–10 males from different litters were used. The whole experiment from F0 to F3 breeding was performed twice. Thus, animals from each generation were derived from two randomly chosen independent treatments. F1 and F3 generation male offspring from the treated and control groups were euthanized at day 60. In this study, we followed paternal lineage because the prostate is a male organ. There are also technical difficulties with the maternal lineage due to the limited number of oocytes to follow maternal inheritance and the requirement for a large amount of material for the assessment of several markers.

A schematic plan of the experiments is shown in Figure S1. F1 and F3 progeny mice were studied at the age of 2 months; at this age, the prostate is functional, and the mice are sexually mature. In this study, we analyzed anterior prostate morphology by staining paraffin sections with hematoxylin and eosin in forty-three F1 and thirty-seven F3 males. In total, 80 male anterior prostates were analyzed. We analyzed the proliferation marker PCNA and phosphorylated histone H3 at serine 10 (PHH3) in prostate paraffin sections. The chromatin histone modifiers HDAC1 and KDM5A and the oncology markers Ki-67 and BRD4 were analyzed via immunofluorescence in frozen prostate sections. We analyzed the histone markers H3K4me3 and H4Ac by purifying the histones and analyzing them via Western blotting. We determined the expression of the genes known to encode proteins important for prostate function and development via RT-qPCR. We analyzed histone H3K4me3 levels at the promoters of the genes via chromatin immunoprecipitation, followed by qPCR. Finally, we analyzed the DNA methylation state of the genes important for prostate development via our previously published datasets.

### 2.2. Direct Exposure to Thiacloprid Induces Epithelial Hyperplasia in the Anterior Prostate

**Figure 1:**
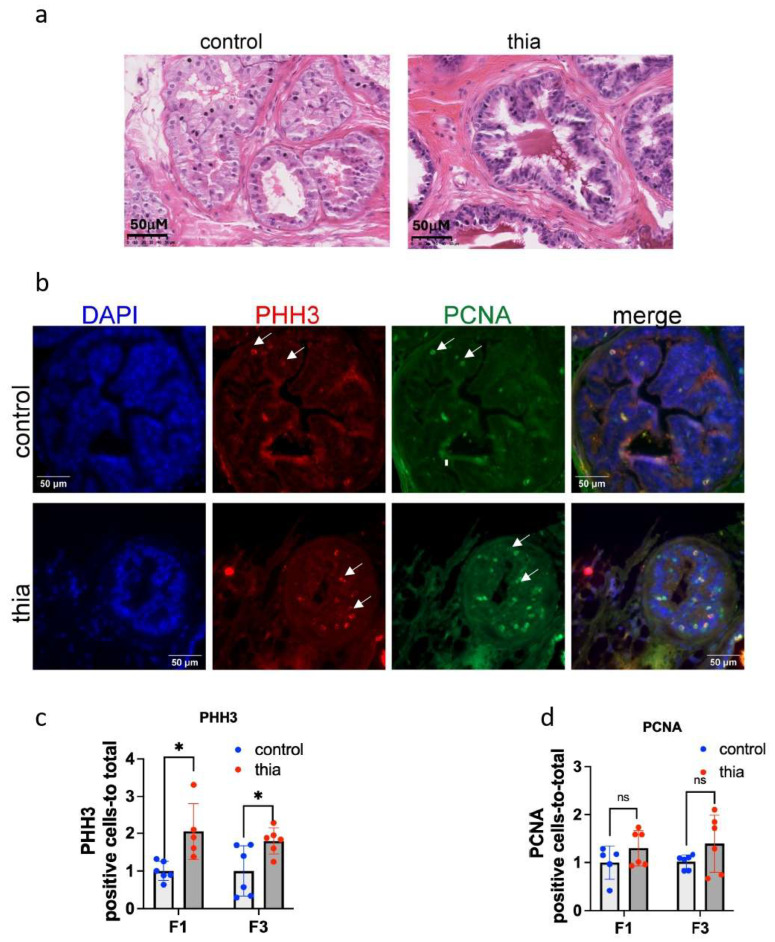
The prostate of *thia*-exposed male mice has a greater number of epithelial hyperplasia and an increase in the expression of proliferation markers. (**a**) Representative images of prostates from control (left) and *thia*-derived (right) mice. The prostate was embedded in paraffin, and 5 mM sections were stained with H&E. (**b**) Representative images of the prostate coimmunostained by PCNA and phosphorylated histone H3 at Serine 10 (PHH3). (**c**) Quantitative analysis of PHH3-positive cells and (**d**) PCNA-positive cells. The PHH3 and PCNA plots in the figure represent the average values of the positive cells per area +/− standard deviation. F1, *n* = 6 control; *n* = 6 treatment; F3, *n* = 6; control, *n* = 6; * *p* < 0.05; Mann–Whitney test. In these and other plots, control groups have individual values in blue, treated groups in red. White arrows show PHH3 or PCNA-positive cells. ns > 0.05.

To identify the effects of thiacloprid on prostate morphology, paraffin-embedded prostate sections were stained with hematoxylin and eosin (H&E), and images were acquired using a digital NanoZoomer scanner. In our analysis, epithelial hyperplasia was identified in the anterior prostate, characterized by regions with a high density of rapidly proliferating epithelial cells forming so-called pseudostratified layers. The cells in the prostate exhibiting epithelial hyperplasia (EPH) display a stratified arrangement with dark, prominent nuclei and nucleoli. Representative images of control prostates and those exposed to epithelial hyperplasia in our study are shown in Figure 1a. In normal prostates, we observe monolayer cells arranged irregularly around the lumen. In prostates with epithelial hyperplasia, we observed regions with densely packed, cylindrical cells that have dark nuclei and prominent nucleoli and occasional pseudo layers. The percentage of prostates with epithelial hyperplasia (EPH) was analyzed in both F1 and F3 generations. In this study, the epithelial hyperplasia phenotype was observed in 5.9% of control group samples (1 out of 17 prostates) and in 27% of the F1-exposed group samples (7 out of 26 prostates) (*p* = 0.08, Chi-square test) Table 1. In the F3 group, 7.1% of control prostates (1 out of 14) and 13% of F3-derived prostates (3 out of 23) exhibited the epithelial hyperplasia phenotype (*p* = 0.6, Chi-square test) (Table 1). Thus, our analysis revealed that gestational exposure to thiacloprid tended to increase the occurrence of hyperplasia in the prostate of directly exposed F1 males, whereas no significant increase was detected in indirectly exposed F3 males.

### 2.3. Proliferation Marker Analysis Revealed an Increase in a Marker of Mitosis

To determine the capacity of prostate cells to proliferate, we carried out a marker analysis. We chose to analyze the expression of PCNA, a marker of cells that are in the early G1 phase and S phase of the cell cycle. PCNA helps in DNA repair during DNA replication [35]. The level of histone H3 phosphorylation on serine 10 (PHH3) is correlated with chromosome condensation; thus, the phosphorylation state of Ser10 is a marker of mitosis [36]. We prepared paraffin sections and coimmunostained them with antibodies against both markers, as described in the Materials and Methods section. Our analysis revealed that the proteins were localized in the nucleus; PCNA was detected throughout the nucleus, and PHH3 appeared as several bright dots in the nuclear periphery (Figure 1b). For both antibodies, we counted several positive cells per prostate area. The PHH3 marker significantly increased in both the F1 and F3 prostates by 2.1 and 1.8 times, respectively (Figure 1c). The quantitative analysis of PCNA revealed increases in F1 and F3 by 1.3 and 1.4 times, respectively (Figure 1d); however, the changes did not pass a significant cutoff threshold. Thus, our analysis of mitosis markers revealed an increase in the prostates of F1 mice, which is consistent with the tendency to increase the epithelial hyperplasia in these mice.

### 2.4. The Oncogenesis Marker Analysis Revealed an Increase in F1 Prostates

**Figure 2:**
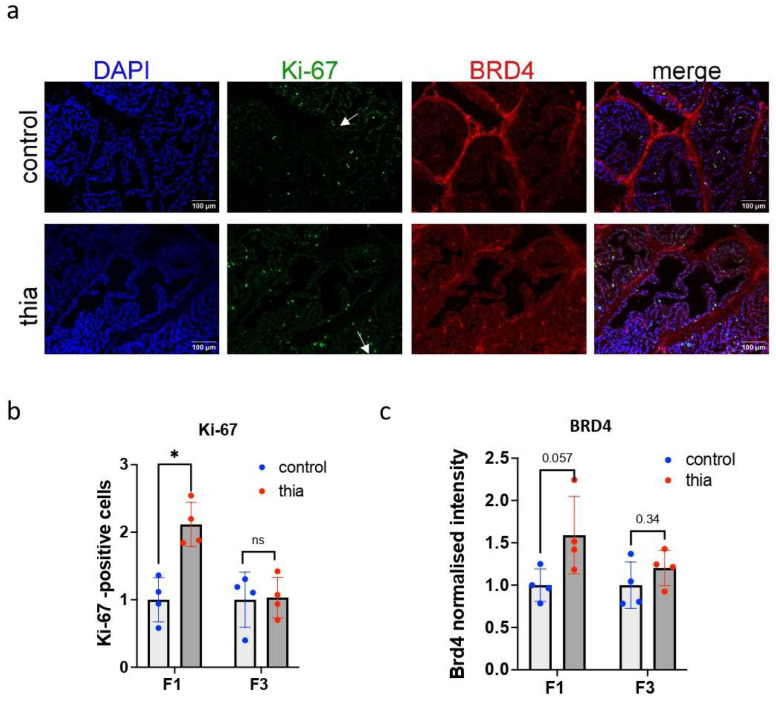
Oncology marker analysis of the prostates of male mice. (**a**) Representative images of prostates from control mice (top) and *thia*-derived F1 mice (bottom) immunostained with Ki-67 (green) and BRD4 (red). Quantitative analysis of the signal intensity of (**b**) Ki-67 and (**c**) BRD4. All plots in the figure represent average values +/− standard deviations. *n* = 4 control, *n* = 4 treatment in F1 and F3, * *p* < 0.05, Mann–Whitney test. The white arrow shows Ki-67-positive cells. ns > 0.05.

Because we observed an increase in the expression of mitosis markers, we verified whether cells could have alterations in oncogenesis markers. We analyzed the protein expression of the marker Ki-67 (also known as MKI67), which is a cellular marker for proliferation [37]. Ki-67 remains active during the G1, S, G2, and M phases of the cell cycle [38] and is also an accepted hallmark of oncogenesis. We also included an analysis of bromodomain-containing protein 4 (BRD4), which is an epigenetic reader that regulates gene transcription through binding to acetylated chromatin via bromodomains [39]. This marker’s expression is also abundant in cancer [40]. To proceed with the experiment, we prepared frozen prostate sections, as Ki-67 and BRD4 antibodies work better in frozen sections. We performed immunostaining in the F1 and F3 prostates as described in the Methods section. Ki-67 was highly intense in some cells, and BRD4 was detected throughout the nucleus (Figure 2a). We analyzed Ki-67-positive cells per prostate area. In F1, the number of Ki-67-positive cells increased significantly by 2.1 times, but no significant changes were detected in F3 (Figure 2b). In F1, BRD4 tended to increase by 1.6 times (*p* = 0.057), but no significant increase was detected in F3 (Figure 2c).

In summary, our analysis revealed that gestational exposure to thiacloprid led to an increase in the proliferation marker Ki-67 in F1 but not in F3. Although BRD4 expression showed a trend toward an increase in F1, this change was not statistically significant. These results suggest that gestational exposure to thiacloprid may influence cell proliferation in F1. This increase in cell proliferation is consistent with a higher incidence of epithelial hyperplasia in F1, but it is not linked to malignant transformation. The oncology markers were not sufficiently assessed for the estimation of the risk of cancer.

### 2.5. RNA Expression Analyses Revealed Increased Expression of Genes Related to Hormonal Pathways, Cellular Proliferation, and Epigenetic Regulation

**Figure 3:**
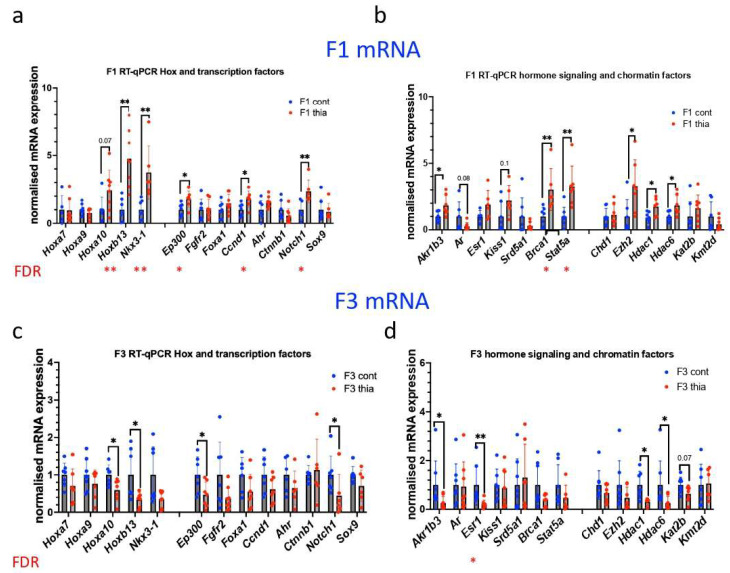
Effects of gestational thiacloprid exposure on gene expression in the prostate. (**a**,**b**) Gene expression analysis of the F1 prostate and (**c**,**d**) the F3 prostate. All plots in the figure represent average values of the fold change +/− standard deviation; *n* = 6 for the control and *thia* groups in F1 and F3; * *p* < 0.05, ** *p* < 0.01, Mann–Whitney test, significant FDR values are shown under the graph, * *p* < 0.05, ** *p* < 0.01.

To gain insight into potential molecular mechanisms that are associated with the phenotypic changes that were observed in these derived males, we carried out RT-qPCR analysis using prostate tissue from F1 and F3 males. We performed a search for the genes that play a role in the prostate via the gene ontology program AMIGO, and we sought a gene list that corresponds to the term “prostate development” (https://amigo.geneontology.org/amigo) accessed on 1 January 2022. Fifty-two genes were identified (Table S1). We added some targets on the basis of our previous research on prostate tissue [41]. We extracted RNA from prostates and prepared cDNA as described in the Methods section. We analyzed the expression of genes related to transcription, hormonal signaling, and chromatin factors (Figure 3a–d). The housekeeping gene *Rpl37a* was used for normalization, as it was reproducible across all of the samples.

In F1 anterior prostates, quantitative analysis revealed that *Hoxb13* and *Nkx3-1* expression increased by 4.8 and 3.7 times, respectively. *Hoxa10* tended to increase in expression by 2.4 times. Both *Ep300* and *Ccnd1* increased in expression by 1.8 times and *Notch1* increased expression by 2.4 times. In addition, *Akr1b3*, *Brca1*, and *Stat5a* presented increases in expression by 1.8, 3.0, and 3.3 times, respectively. *Kiss1* tended to increase 2.2 times. We also assessed the impacts of these factors on the expression of genes encoding chromatin factors. We determined that in F1, the expression of *Ezh2, Hdac1*, and *Hdac6* increased by 3.2, 1.7, and 1.8 times, respectively. In contrast to most of the analyzed genes, the expression of the *Wnt* signaling pathway gene *Ctnnb1* tended to decrease by 0.6 times.

In F3, *Hoxa10*, *Hoxb13*, *Ep300*, and *Notch* expression decreased by 0.6, 0.3, 0.5, and 0.5 times, respectively. *Fgfr2*, *Foxa1*, and *Ccnd1* tended to decrease by 0.4, 0.6, and 0.6 times, respectively. In addition, *Akrb3*, *Esr1*, *Brca1*, *Hdac1,* and *Hdac6* presented decreases in expression by 0.3, 0.2, 0.3, 0.3, and 0.6 times, respectively. We checked the possible origin of the tested genes using human single-cell sequencing data. Notably, a large number of genes are of epithelial origin (*Hoxa9*, *Sox9*, *Kiss1*, *Nkx3-1*, *Foxa1*, *Ar*, *Hoxb13*); a few of the genes are expressed in the immune system and in basal cells, suggesting that we cannot exclude the possibility that the observed increase in gene expression could also be related to an increase in the number of epithelial cells.

In summary, our analysis of RNA expression in the prostates of F1 and F3 offspring revealed that gestational exposure to thiacloprid altered the expression of genes associated with prostate morphology, cellular proliferation, chromatin factors, and genes encoding proteins of the hormonal signaling pathway. Most of the analyzed genes were upregulated in F1 and downregulated in F3.

### 2.6. Analysis of the Major Regulatory Histones Revealed the Global Impact on H3K4me3 Levels in F1 and F3 Prostates

**Figure 4:**
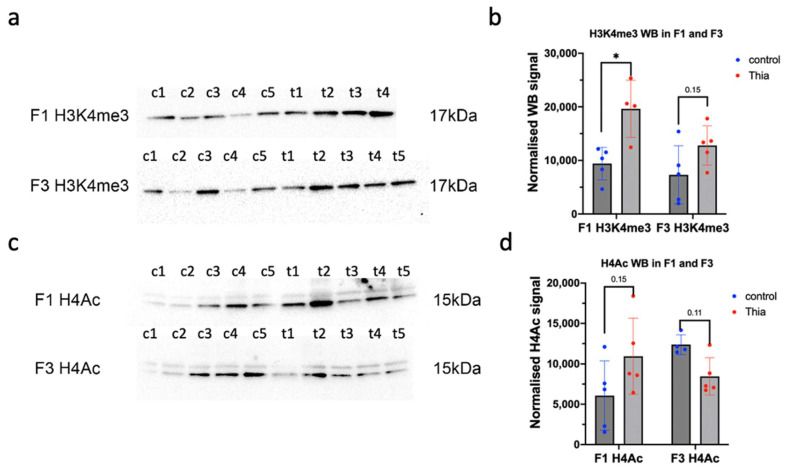
Analysis of purified histone proteins via WB. (**a**) Representative image of H3K4me3 in F1 and F3; c1–c5 represent the control and t1–t5 represent the *thia* groups. (**b**) Quantitative analysis of H3K4me3. (**c**) Representative image of H4Ac blot in F1 and F3; c1–c5 represent the control and t1–t5 represent the *thia* groups. (**d**) Quantitative analysis of H4Ac. All plots in the figure represent average values +/− standard deviations. * *p* < 0.05, nonparametric Mann–Whitney test.

Next, we analyzed the effects of thiacloprid on epigenetic regulation. To this end, we chose to investigate the effects of thiacloprid on H3K4me3, major regulatory marks. H3K4me3 marks are abundant in regions with open chromatin. We also analyzed the H4 acetylation level because this marker is associated with chromatin compaction and it is abundant in cancer cells. We extracted histone proteins and performed Western blot analysis. We revealed that H3K4me3 levels had increased by 2.0 times in F1 and H3K4me3 tended to increase by 1.8 times in F3 (Figure 4a,b). Histone H4 acetylation levels tended to increase 1.9 times in F1 offspring (*p* = 0.15). In contrast to H3K4me3, the H4 acetylation level tended to decrease 0.7 times (*p* = 0.11) in F3 (Figure 4c,d).

Thus, our analysis revealed that major regulatory marks were increased in F1, suggesting that exposure to thiacloprid has a global impact on the processes associated with gene expression.

### 2.7. H3K4me3 Levels in the Gene Promoters Showed Similar Alterations to Gene Expression Changes

**Figure 5:**
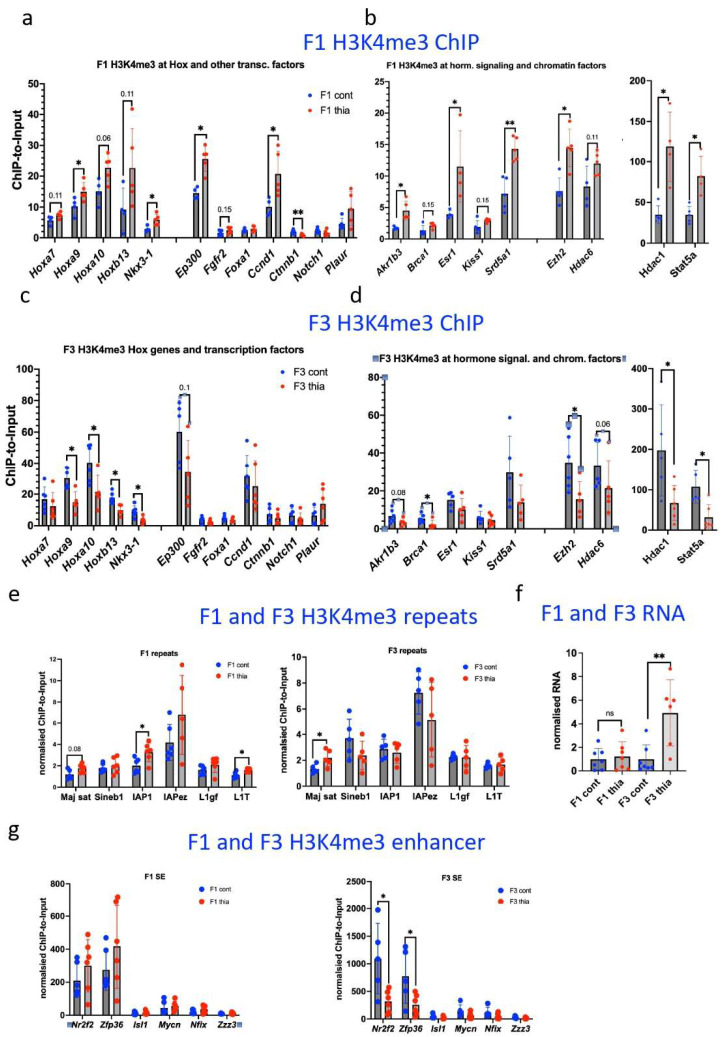
Effects of gestational thiacloprid exposure on H3K4me3 histone occupancy. (**a**) Analysis of H3K4me3 occupancy at the promoters of homeobox, genes encoding transcription factors (**b**) at the promoters of hormone signaling, and chromatin factors encoding genes in F1. (**c**) Analysis of H3K4me3 occupancy at the promoters of homeobox and genes encoding transcription factors, (**d**) at the promoters of hormone signaling, and chromatin factors encoding genes in F3. (**e**) Analysis of H3K4me3 occupancy at repeated elements in F1 and F3. (**f**) Analysis of satellite RNA expression in F1 and F3. (**g**) F1 and F3 H3K4me3 enhancer occupancies. F1: *n* = 5 controls, *n* = 6 treatment; F3: *n* = 5 controls, *n* = 6 treatment. All plots in the figure represent average values +/− standard deviations. * *p* < 0.05, ** *p* < 0.01, Mann–Whitney test. ns > 0.05.

Because the H3K4me3 marker was altered in F1 and tended to change in F3, we performed ChIP–qPCR to confirm the role of this marker in gene expression changes. We designed primers at the regions of the genes that were chosen for RT–qPCR analysis via the primer-blast program from https://www.ncbi.nlm.nih.gov/tools/primer-blast/index.cgi?LINK_LOC=BlastHome, accessed on 1 January 2022, and our previously published datasets of H3K4me3 in the prostate [41]. As a control for the ChIP background, we used the region of the *Gapdh* gene, which is located distal to the promoter. We performed ChIP and analyzed the quantity of immunoprecipitated material compared with that of a starting material named Input, as described in the Materials and Methods section. The quantitative analysis revealed that the histone H3K4me3 changes were consistent with the gene expression changes (Figure 5d). For example, H3K4me3 occupancy at *Hoxa9* was elevated by 1.5 times. H3K4me3 levels at the promoters of *Hoxa10* and *Hoxb13* tended to increase by 1.6 and 2.8 times, respectively. *Ep300* and *Ccnd1* increased H3K4me3 levels by 1.8 and 1.9 times, respectively. A significant increase in H3K4me3 was also detected at *Akr1b3* (2.2 times), *Esr1* (3.3 times), *Srd5a1* (2.0 times), and *Stat5a* (2.4 times). Gene encoding chromatin factors had elevated levels of H3K4me3 at their promoters upon exposure to thiacloprid, e.g., *Ezh2* (1.7 times), *Hdac6* (1.4 times), and *Hdac1* (3.4 times). Notably, *Ctnnb1* decreased H3K4me3 by 0.5 times.

In F3, we detected a decrease in H3K4me3 at the promoters of the analyzed genes. Specifically, we noted significant decreases in that marker in the promoter of *Hoxa9* (0.5 times), *Hoxa10* (0.5 times), *Hoxb13* (0.6 times), and *Nkx3-1* (0.4 times). We determined that the promoter of Ep300 H3K4me3 tends to decrease (0.6 times). The H3K4me3 levels at *Brca1* and *Ezh2* decreased by 0.4 times. *Akrb13, Esr1, and Hdac6* tend to have decreased H3K4me3 levels by 0.5, 0.7, and 0.6 times, respectively. *Hdac1* and *Sta5a* both decreased H3K4me3 levels by 0.3 times.

Our analysis of H3K4me3 showed that there are similar alterations in *thia*-derived males regarding gene expression, suggesting that thiacloprid exposure induces epigenetic alterations that probably impact transcription factor binding to gene promoters. However, in F3, despite the increase in H3K4me3 histone protein levels, most of the analyzed genes presented a decreased level of H3K4me3, suggesting that some other regions not analyzed by ChIP–qPCR could contribute to the global level of H3K4me3. We thought that repeated regions could contribute to the observed increase in H3K4me3. To test our hypothesis, we analyzed the presence of repetitive regions in H3K4me3 using the ChIP-qPCR method. We chose to analyze the level of H3K4me3 in retroelements and satellite DNA. We determined that H3K4me3 has increased at major satellite regions, IAP, and L1 retroelements in F1. In F3, we determined an increase in H3K4me3 at major satellite regions but no significant changes at other tested elements (Figure 5e). Satellite DNA is an array of noncoding tandem repeat sequences that are most concentrated in the centromere regions of chromosomes but can also be interspersed throughout the chromosome or localized near the telomeres. Because satellite RNAs play a role in the structure of chromatin, we checked the level of expression of satellite RNAs. We observed that in F3, there is an increase in major satellite expression (Figure 5f). We also checked the H3K4me3 levels at enhancer elements. We chose to analyze super-enhancer regions that have a higher level of H3K4me3 marks due to the possibility of binding many transcriptional factors. We did not detect a statistically significant difference in F1, but, in F3, a decrease in H3k4me3 in the vicinity of *Nr2f2* and *Zfp36* was determined (Figure 5g). Notably, these two regions had a very high ChIP-to-Input ratio, suggesting a possible role of these alterations in H3K4me3 markers on gene expression, as H3K4me3 markers are enriched at transcriptionally active chromatin regions.

### 2.8. Increased HDAC1 Levels Were Detected in the F1 Prostate

**Figure 6:**
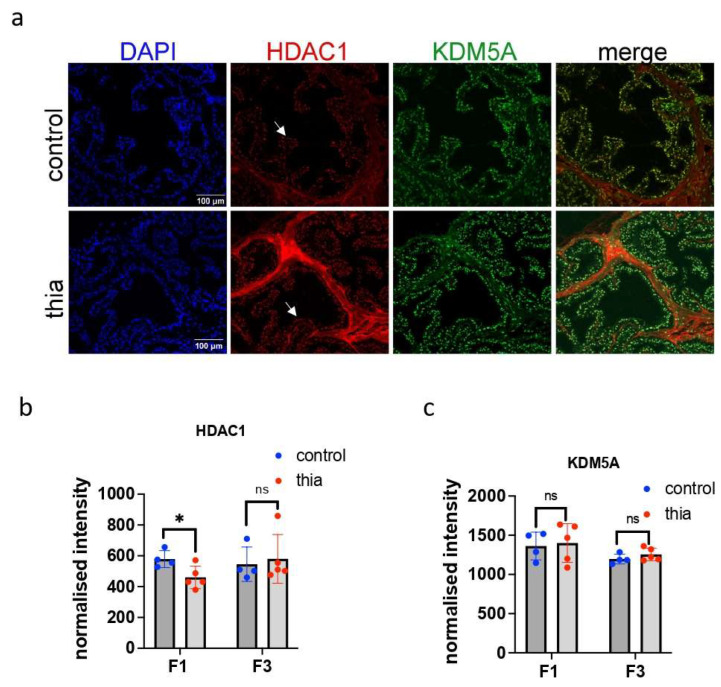
Chromatin marker analysis of the prostate of male mice. (**a**) Representative images of prostates immunostained for KDM5A (green) and HDAC1 (red) from control (top) and treatment (bottom) mice. Quantitative analysis of the signal intensity of (**b**) HDAC1 and (**c**) KDM5A in F1 and F3 males. All plots in the figure represent average values +/− standard deviations. F1 and F3, *n* = 4 for control and treatment groups, * *p* < 0.05, Mann–Whitney test. All plots in the figure represent average values +/− standard deviations. The mice were sacrificed at 2 months of age. White arrows show HDAC1-positive cells. ns > 0.05.

Because class I HDAC levels are increased in anterior prostate cancers and their aberrant expression is correlated with decreased tumor suppressor activity and drug resistance [42,43], we analyzed HDAC1 levels in the F1 and F3 anterior prostates via immunofluorescence. Histone deacetylase 1 (HDAC1) is a KDM5A-dependent factor, and these two factors coregulate their downstream genes [44]. KDM5A is also involved in the transcriptional regulation of *Hox* genes and plays a role in tumor progression [45]; thus, we analyzed the protein level of KDM5A, as well. We prepared frozen sections and fixed them with paraformaldehyde, and we performed immunofluorescence analysis of the intensity of these markers per area of prostate epithelial cells. Our immunofluorescence staining revealed that these markers have bright staining in the nucleus and they are colocalized (Figure 6a). In F1, we detected a significant increase in the HDAC1 level (1.4 times) (Figure 6b). In contrast to F1, we observed a slight but not significant decrease (0.9 times) in HDAC1 in the F3 anterior prostate. KDM5A analysis revealed no significant changes in the prostates, suggesting the alteration in HDAC1 is not dependent on KDM5A (Figure 6c).

In summary, we observed an increase in HDAC1 levels in the F1 prostate, and this increase in HDAC1 intensity is consistent with elevated *Hdac1* gene expression and increased H3K4me3 levels at the promoter of HDAC1. The intensities of KDM5A in F1 and F3, as well as HDAC1 in F3, were not significantly modified.

### 2.9. Sperm DNA Methylation Analysis Revealed a Potential Impact of Thiacloprid on the Regions Important for Prostate Development

To assess whether sperm DNA methylation could be associated with gene expression changes, we reanalyzed the sperm DNA methylation of F1 and F3 males from our previously published datasets [17]. We examined DNA methylation at *Hdac1* and determined that in the vicinity of ~20 kb, there is a DNA methylation marker that tends to decrease in F1 and increase in F3 (Figure S2a,b), suggesting a possible link between paternal sperm DNA methylation and histone H3K4me3 in the prostate at the *Hdac1* gene.

**Figure 7:**
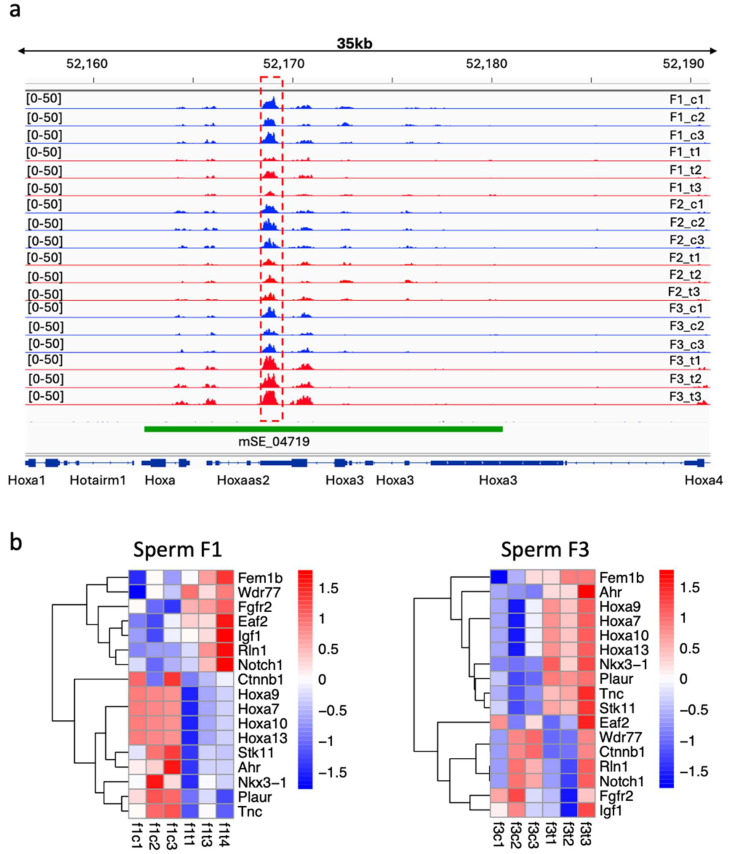
DNA methylation analysis of the genes that play a role in prostate development in the sperm of F1 to F3 mice. (**a**) Plots of differentially methylated regions overlapping the *Hoxa3* gene; the signal range is indicated in brackets; the differential peak is marked by the red dashed box. (**b**) Heatmap of DMRs in the sperm of F1 and F3 males. MeDIP counts that have fold change > 1.5 and FDR < 0.1 were log-transformed and plotted in R via Heatmap; f1c1–f1c3 are F1 controls; f1t1–f1t3 are F1 *thia*-derived samples; f3c1–fc3 are F3 controls; and f3t1–f3t3 are F3 *thia*-derived samples. The sequencing analysis was performed using sperm DNA and a minimum of 3 replicates for each group from F1 to F3.

We also performed DNA methylation analysis of genes that are important for prostate development. We used the gene list determined from the AMIGO database as “prostate development” genes (Table S3). We verified the presence of DNA methylation markers near these genes in our sperm datasets from F1, F2, and F3 males and compared the samples from *thia-*derived males and the control. The analysis revealed that in sperm, several genes have alterations in DNA methylation in F1 and F3 males. We observed DNA methylation markers in the region near the *Hoxa3* gene (Figure 7a). This region also overlaps with the superenhancer element mSE_04719, which was found to be active in the embryonic brain. In the prostate, there are also neuroendocrine cells that have a lot of similarity with neuronal cells, as neuroendocrine cells in both tissues produce and release hormones into the blood. Thus, *Hoxa3* could be driven by a super-enhancer active in the embryonic brain and in the neuroendocrine cells of the prostate. However, whether it is active in developing or adult prostates was not assessed. Notably, the region was found to have decreased DNA methylation in F1 and F2 sperm but increased methylation in F3 sperm. In addition to *Hoxa3*, some other regions were found to have altered DNA methylation in the sperm, including regions near the *Stk11*, *Ahr*, *Nkx3-1*, *Plaur*, *Tnc*, *Wdr77*, *Fgfr2*, *Eaf2*, and *Igf1* genes (Figure 7b).

Our study suggests that differentially expressed genes are associated with altered DNA methylation in the prostate; however, the direct link between these alterations in gene expression was not established.

## 3. Discussion

### 3.1. Effects of Thiacloprid on F1 and F3 Generations

We determined the tendency toward increased epithelial hyperplasia in F1 prostates and elevated expression of mitosis (PHH3) in F1 mice. Our data are consistent with previous observations of other authors, who noted that endocrine-disrupting compounds such as BPA [46] or phthalates [47] could induce the appearance of epithelial hyperplasia in the prostate. In these studies, the exposure periods were gestational or early neonatal, suggesting that early development is critical for the establishment of cell lineages specific to the prostate. Indeed, alterations in developmental genes of the prostate could impact the normal development of the prostate in adult animals. Thus, it is likely that gestational exposure to thiacloprid induces some alterations in the F1 generation, causing an increase in proliferation, based on analysis of markers. However, the effects were not detected in F3, suggesting that most of the effects induced in F1 were not transgenerational but intergenerational.

### 3.2. Thiacloprid-Induced Epigenetic Effects

In this study, we analyzed H3K4me3 through WB and ChIP–qPCR. The H3K4me3 global level increased in both generations, which could be due to an increased level of mitosis resulting from a significantly increased level of PHH3, a marker of mitosis, in F1 and F3. A high level of H3K4me3 in the prostate was shown in our previous transgenerational study [41], and we observed an elevated level of proliferation markers and an increased rate of epithelial hyperplasia. The increase in proliferation changes the chromatin structure to a more accessible configuration [48]. H3K4me3 markers are recognized by many transcription factors and other chromatin readers [49]; therefore, H3K4me3 markers create a platform for increased transcription. For example, in the developing prostate, endocrine-disrupting compounds induce MLL1/KMT2A activation, which causes increased H3K4me3 levels in genes associated with prostate cancer, and, importantly, the elevated expression of these genes persists in adulthood [50]. Elevated H3K4me3 levels were detected in hormone-resistant prostate cancer (HRPC) [51].

We observed that WB and ChIP-qPCR data were not consistent in F3. We noted that H3K4me3 markers at major satellite sequences localized to the centromeric region of the chromosomes were increased in F1 and F3. It has been shown that satellite sequences are not transcriptionally inert; the transcribed RNA contributes to the structural organization of heterochromatin [52]. Thus, an increase in H3K4me3 may contribute to the structural and organizational compactization of chromatin. However, the current data do not fully resolve the discrepancy, and the mechanisms that are responsible for the difference have not been studied.

In contrast to H3K4me3, H4Ac tended to increase in F1 and decrease in F3 in our study, suggesting that exposure to *H4Ac* creates more accessible chromatin in F1 than in F3. Notably, although H4Ac tends to increase in F1 offspring, we observed that the HDAC1 protein was abundantly present in our F1 offspring. An abundance of HDAC1 was previously observed in malignant epithelial nuclei in prostate tissue [53]. The differential HDAC expression in epithelial and stromal cells may play important roles in the progression of prostate cancer [54]. We did not measure the HDAC1 level in stromal tissues, but it is possible that stromal and epithelial cells could have differential HDAC levels.

We determined that DNA methylation of several genes essential for prostate development was altered. Indeed, *thia* exposure overlaps with the embryonic period of the somatic-to-germline transition when DNA methylation is erased and re-established de novo. The perturbation of the activity of DNA methyltransferases is induced by numerous environmental factors [55]; thus, it is conceivable that some alterations in DNA methylation in our study were established due to the perturbation of de novo methyltransferases, such as DNMT3A. Some of these alterations could be preserved by subsequent generations. However, most DNA methylation markers are erased after fertilization. Only limited regions resist reprogramming. For example, some IAP-containing regions avoid reprogramming [56,57]. In our previous *thia-*exposed testis study, we showed that near the *Isl1* region, DNA methylation decreases in F1 and F3, and this region overlaps with the IAP retroelement. Recently, the role of ISL1 was demonstrated in prostate cancer. *Isl1* knockdown reduces androgen receptor (AR) activity and leads to reduced cell growth in castration-resistant prostate cancer [58], suggesting a potential role for *Isl1* in prostate proliferation in our study. The role of *Isl1* in pathology induced by thiacloprid was not evaluated in this study. Besides *Isl1* in our previous study, we showed that nearly 30 regions had similar directions in changes in F1 and F3, including regions near *Cdk5rap2*, *Large1*, *Abhd18*, *Grin3a*, *Sox2*, *Pdgfa*, Zfp36l2, *Inha*, *Vrk1*, and *Hif1a* [17]. These regions could play the role of master regulators as they encode for important transcriptional factors.

We also noted that alterations in our studies in F1 and F3 epigenetic marks had opposite effects. We cannot simply explain this phenomenon; we suggest that some unknown mechanisms compensate for the previously induced epigenetic alterations in F3 males. Similar opposite effects were observed in our previous study in prostates exposed to chlordecone [41]. We suggest that compensatory effects could be promoted during fertilization. The chromatin rearranged after fertilization to form a functional embryonic genome. In the paternal genome, chromatin nucleosome domains are re-established. The sequencing of early embryos revealed that the maternal genome started to reduce the size of the broad H3K4me3 peaks after fertilization, while paternal H3K4me3 markers are detectable at very low levels only in some regions [59,60], suggesting that H3K4me3 markers of both parental genomes were rearranged in new embryos. A recent study revealed that in zebrafish, there are regions named “placeholders”, which contain the histone H2A variant H2A.Z and H3K4me1; these regions occupy all regions lacking DNA methylation in both sperm and embryos and reside at promoters encoding housekeeping and early embryonic transcription factors [61]. Thus, other histone modifications, such as H2A.Z, may play a role in the rearrangement of histone and DNA methylation markers after fertilization and the establishment of new patterns in new embryonic tissues.

In addition to histone markers, we should also consider the possible role of noncoding RNA in the phenotypes. The altered ncRNA could be generated and packed in the sperm of directly exposed F1 and F2 but not in F3.

### 3.3. The Possible Impact of the Withdrawal of Thiacloprid on the Ecosystem

In 2018, neonicotinoids were banned in Europe. However, recovery is not that fast. A recent study showed that banning neonicotinoids does not guarantee immediate recovery of the species [62]. Insect-eating bird populations in France appear to be making a tentative recovery after a ban on bee-harming pesticides, according to a study that examined wildlife in Europe after the banning of neonicotinoids [62]. We believe that the persistence of neonicotinoids in soil may reduce recovery. However, there is some hope that the current, even small increase in the bird population will continue in the future at a higher rate as soon as neonicotinoids are degraded from the soil.

## 4. Materials and Methods

### 4.1. Ethics Statement

All experimental procedures and protocols were approved by the Ethics Committee before starting the experiments. All experimental procedures involving animals were authorized by the Ministry of National Education and Research of France (Number APAFIS#17473-2018110914399411 v3). The animal facility used for the present study is licensed by the French Ministry of Agriculture (agreement D35--238--19). All experimental procedures followed the ethical principles outlined in the Ministry of Research Guide for Care and Use of Laboratory Animals and were approved by the local Animal Experimentation Ethics Committee (C2EA-07). All methods were performed under the ARRIVE guidelines. Mice were purchased from Janvier Lab. Animals were allowed to adapt for one week before the experimental procedure. Animals were housed in a dedicated animal facility in standard cages and under a regular daily light cycle. The personnel ensured that temperature and relative humidity fell within the recommended ranges. Husbandry was performed by placing together males and females, and, in the morning, the vaginal plugs were rechecked. The day of vaginal plug detection was considered embryonic day 0.5. The animal started to be treated through gavage with 6 mg/kg/day of thiacloprid suspended in oil or by vehicle from E6.5 to E15.5; this window corresponds to the formation of precursors of gametes. Oral intake was chosen due to the way most species, including humans, are contaminated by pesticides. The animals were euthanized by placing them in a carbon dioxide (CO_2_) chamber. All procedures were authorized by the Ministry of National Education and Research of France; the authorization number is #17473-2018110914399411 v3. All euthanasia procedures were performed according to annex IV of Law 2013–118 issued by the Ministry of Agriculture, Food and Forestry of France on 1 February 2013.

### 4.2. Mouse Treatment and Dissections

Outbred Swiss mice (RjOrl) not genetically modified were used for all of the experiments. The exposure window was limited to a period between E6.5 and E15.5, which corresponds to the somatic-to-germline transition (SGT) [63]. Thiacloprid (*thia*) was administered at a dose of 6 mg/kg body weight/day. All treatments were performed in the animal procedure room. *Thia* is distributed throughout the body following exposure and can pass through the placental barrier according to previous studies [64]. In our study, *thia* was suspended in olive oil and administered via oral gavage at a dose of 6 mg/kg body weight/day in a volume of 150 μL. Oral intake was chosen due to the way humans are contaminated by pesticides. The control mice were treated with the same volume of oil. Male progeny of F0 females were defined as F1, and these males were crossed with nonlittermate untreated females to give rise to the F2 generation. Similarly, the male progeny of F2 were crossed with nonlittermate untreated females to give rise to the F3 generation. The oil-treated control animals were processed and crossed in the same way as the *thia*-treated control animals. Because we were particularly interested in the transgenerational inheritance of prostate pathologies, we analyzed the anterior prostate of the F1 directly exposed and non-exposed F3 generation males. The prostates from the F1 and F3 generations were analyzed when the animals were 2 months old. For morphology evaluations, male progeny from a minimum of 8 nonrelated treated females (F0) or untreated (F3) females were used for each group; in total, 80 animals were analyzed. For most of our experiments, we used a minimum of 4–8 biological replicates derived from independent, nonrelated treated mothers. The sample size was determined by the variation and reproducibility of each experiment.

### 4.3. Prostate Morphology Analyses

To study prostate morphology, anterior prostates from F1 and F3 control mice and the corresponding treated groups were fixed in Bouin’s solution for 24 h, washed in PBS and in 70% ethanol, dehydrated, and embedded in paraffin. Five-micron sections of the entire prostate were cut, and the sections were placed on glass slides. The sections were deparaffinized and stained with H&E. Pictures were taken with a digital slide scanner (NanoZoomer, HAMAMATSU, 812 Joko-cho, Higashi-ku, Hamamatsu City, Japan), and analyzed via NDPview software (v2.7.25) by two independent researchers. We analyzed 43 prostates in F1 and 37 in F3, for a total of 80 individual anterior prostates.

### 4.4. Immunofluorescence of Prostate Tissue in Paraffin Sections

For immunostaining, the paraffin blocks of the prostates from the F1 and F3 control and treated groups were used. The sections were cut with a microtome (Microm HM 355 S, Thermo Fisher Scientific, Francheville, France) at a thickness of 5 μm. The sections were deparaffinized and rehydrated, and the epitopes were unmasked in 0.01 M citrate buffer, pH 6, at 80 °C for 45 min. The slides were blocked and incubated with rabbit anti-PHH3 (1:500, 07-327, Merck Millipore, Darmstadt, Germany) and mouse PCNA (1:500, ab29, Abcam, Cambridge, USA) antibodies. The sections were incubated with primary antibodies in PBS-T overnight at 4 °C in a humidified chamber. After being washed in PBS-T, the sections were incubated with an appropriate fluorescent secondary antibody (1:100; AlexaFluor from Invitrogen, Carlsbad, CA, USA) for 1 h in a humidified chamber at room temperature. The sections were counterstained with the Vectashield solution (Eurobio Scientific, Chicago, IL, USA). The images were taken via an AxioImager microscope equipped with an AxioCam MRc5 camera and AxioVision software version 4.8.2 (Zeiss, Oberkochen, Germany) with a 5× or 40× objective lens using the same exposure time (DAPI, 350/442; Alexa Fluor 488, 488/525; Alexa Fluor 594, 594/617). The images were left unprocessed before analysis. For PCNA and PHH3, we quantified positive cells per area and analyzed a minimum of 6 images per slide from at least 6 different anterior prostates via Fiji version 2.9.0 software. The data were averaged and are presented as the relative fluorescence compared with that of the control ± SD. The combined total corrected fluorescence (CTCF) was calculated as a subtraction from the integrated density of the mean intensity of the background area multiplied by the area. The mean intensity of the background was calculated for each image in the area without any cells. The data were averaged and are presented as positive cells per area compared with the control ± SD.

### 4.5. Immunofluorescence of Anterior Prostate Tissue in Frozen Sections

For immunostaining, prostates from the F1 and F3 control and treated groups were embedded in optimal cutting temperature (OCT) and 7 mM sections were cut, dried, and stored at −80 °C before use. The slides were fixed with 4% paraformaldehyde for 8 min at 4 °C. The slides were quenched with 0.1 M glycine in PBS for 5 min and then washed 3 times with PBS. The slides were blocked with 4% BSA in PBS and incubated with rabbit anti-Ki-67 (1:500, ma5-14520, Invitrogen, Waltham, MA, USA) and mouse BRD4 (1:500, sc-518021, Santa Cruz, City of Santa Cruz, CA, USA) or anti-mouse HDAC1 (1:500, sc-81598, Santa Cruz, City of Santa Cruz, CA, USA) and anti-rabbit KDM5A (1:500, ab194286, Abcam, Cambridge, MA, USA) antibodies diluted in DAKO (S2022, Agilent, Santa Clara, CA, USA). The sections were incubated with primary antibodies overnight at 4 °C in a humidified chamber. After 3 washes in 0.4% Kodak Photo Flo in PBS, the sections were incubated with an appropriate fluorescent secondary antibody (1:100; AlexaFluor from Invitrogen) for 1 h in a humidified chamber at room temperature. The sections were counterstained with the Vectashield solution (Eurobio Scientific, Chicago, IL, USA). The images were taken via an AxioImager microscope equipped with an AxioCam MRc5 camera and AxioVision software version 4.8.2 (Zeiss, Oberkochen, Germany) with a 40X objective lens using the same exposure time (DAPI, 350/442; Alexa Fluor 488, 488/525; Alexa Fluor 594, 594/617). The images were left unprocessed before analysis. For Ki-67, we quantified positive cells per area and analyzed a minimum of 6 images per slide from at least 4 different anterior prostates via ImageJ software 2.9.0. For BRD4, KDM5A, and HDAC1, we used the lasso tool in ImageJ, drew the anterior prostate contour around epithelial cells, and measured the fluorescence. Combined total corrected fluorescence (CTCF) was calculated as a subtraction from the integrated density of the mean intensity of the background area multiplied by the area. The mean intensity of the background was calculated for each image, and we used the area without any cells. The data were averaged and are presented as the relative fluorescence compared with the control ± SD or the number of positive cells per area compared with the control ± SD.

### 4.6. RNA Extraction and RT–qPCR

For the analysis of the F1 and F3 prostates, total RNA was extracted from the prostates that were snap-frozen following dissection via the RNeasy plus mini kit (Qiagen, Hilden, Germany) according to the manufacturer’s instructions. The kit includes a DNA elimination step; RNA was treated with a RNase-Free DNase Set (79254, Qiagen, Hilden, Germany). For RT-qPCR analysis, 6 biological replicates from control and F1- and F3-generation males were used. Reverse transcription was performed with 1 µg of RNA via the iScript Reverse Transcription Kit (Invitrogen, Waltham, MA, USA) according to the manufacturer’s instructions. The resulting cDNA was diluted 10 times and used for quantitative PCR. qPCR was performed using a Bio-Rad 384 plate machine with iTaq Universal SYBR Green Supermix (Bio-Rad, 1725124, Hercules, CA, USA). Ct values for *Rpl37*, a housekeeping gene, were used for normalization via CFX Manager software (Bio-Rad CFX Maestro 2.3, version 5.3.022. 1030) provided with the Bio-Rad 384 plate machine. The sequences of primers used for qPCR are shown in Table S3. The data were analyzed and are presented as the mean values of the fold change (FC) compared with the control. A nonparametric Mann–Whitney test was applied to assess statistical significance.

### 4.7. Histone Purification and Western Blot Analysis

Protein samples from F1 and F3 mice of the prostate were prepared via the EpiSeeker Histone Extraction Kit (Abcam, 113476) according to the manufacturer’s protocol. Briefly, mouse prostates were homogenized via a TissueLyser (Qiagen) and centrifuged at 900× *g* for 5 min. The pellets were resuspended in lysis buffer and left on ice for 30 min. After centrifugation, the supernatant fractions containing acid-soluble proteins were transferred to new tubes and balance-DTT buffer was added. The protein concentrations were determined via the Pierce™ 660 nm Protein Assay (ThermoScientific, Waltham, MA, USA). Five micrograms of protein were run on a 4–15% Mini-Protean precast polyacrylamide gradient gel (Bio-Rad, USA) for 1 h, and the proteins were transferred onto ImmobilonPSQ membranes (Merck Millipore, Darmstadt, Germany) via an electroblotter system (TE77X; Hoefer, Holliston, MA, USA) for 2 h. Subsequently, blocking was carried out via the addition of 5% skim milk in PBS-Tween 20 (0.05%) for 1 h. Proteins were detected via rabbit polyclonal anti-trimethyl-histone H3K4me3 (Millipore, 07-473; 1:10,000 dilution) or rabbit polyclonal anti-hyperacetylated histone 4 (Penta, Merck Millipore, 06-946; 1:10,000 dilution). The signal intensities of histone modifications were normalized against the band intensities from the red Ponceau-stained membrane. The data are presented as normalized signals compared with the control +/− SD. The uncut original WB images are provided in Supplementary Figures S3 and S4.

### 4.8. Chromatin Immunoprecipitation and ChIP–qPCR

We performed ChIP using rabbit polyclonal antibodies against H3K4me3 (07-473, Millipore). Equal amounts of material (~one prostate from one mouse) were used and incubated in 1% paraformaldehyde solution for 10 min to crosslink proteins to DNA. One hundred microliters of 1.25 M glycine were added to each sample to quench the unbound paraformaldehyde. The samples were centrifuged, and 1 mL of PBS and two metal beads were added to the pellet, which was homogenized via TissueLyser (Qiagen). The samples were subsequently filtered in a cell strainer, and the resulting solution was pelleted and resuspended in the following buffer: 0.25% (*v*/*v*) Triton X-100, 10 mM EDTA, 0.5 mM EGTA, and 10 mM Tris (pH 8). The samples were centrifuged at 1100× *g* for 5 min at 4 °C, and the pellets containing the cells were resuspended in 300 μL of SDS lysis buffer (1% (wt/vol) SDS, 10 mM EDTA, and 50 mM TrisCl, pH 8) in the presence of a protease inhibitor and 10 mM DTT and incubated for one hour at RT. Chromatin was sonicated in SDS lysis buffer at 60% amplitude for 8 min (20 s on, 20 s off) via a Qsonica 700 sonicator (Q700–110, Newtown, CT, USA) supplied with a 431C2 cup horn; these parameters allowed us to obtain ~300 bp chromatin fragments. After sonication, the samples were centrifuged at 12,800 rpm for 10 min at 4 °C, and the supernatant containing the sonicated chromatin was transferred and diluted in 1.7 mL of the following buffer: 0.01% (1.1% (*v*/*v*) Triton X-100, 1.2 mM EDTA, 16.7 mM TrisHCl, and 167 mM NaCl. A solution containing 20 μL of Dynabeads (10002D, Invitrogen) and 0.7 µL of anti-H3K4me3 antibody (07-473, Millipore) was added to the sample tubes and incubated overnight at 4 °C. Before the antibody and Dynabeads were added, 10 μL of each sample was collected as “Input samples” (starting material). After overnight incubation with Dynabeads and the antibody of interest, the beads were washed 5 min each with the following four buffers: (1) low salt buffer: 0.1% (wt/vol) SDS, 1% (*v*/*v*) Triton X-100, 2 mM EDTA, 20 mM TrisHCl, 150 mM NaCl; (2) high salt buffer: 0.1% (wt/vol) SDS, 1% (*v*/*v*) Triton X-100, 2 mM EDTA, 20 mM TrisCl pH 8, 500 mM NaCl; (3) LiCl buffer: 0.25 M LiCl, 1% (*v*/*v*) Igepal, 1 mM EDTA, 10 mM TrisCl, pH 8, 1% (wt/vol) deoxycholic acid; and (4) TE buffer (two washes). Following the washing steps, the beads were resuspended two times in 50 µL of 1% (wt/vol) SDS and 0.1 M NaHCO_3_ (pH = 9) and incubated at 65 °C for 15 min to elute the precipitated chromatin from the beads. The eluted chromatin was subsequently reverse crosslinked by adding 9 µL of 5 N NaCl and incubating at 65 °C for 4 h. The proteins were subsequently removed by adding 1 µL of 20 mg/mL proteinase K and incubating the samples for 1 h at 45 °C. The precipitated DNA was purified via a MiniElute Reaction Clean-Up Kit (Qiagen), and the DNA concentration was measured via the QuantiFluor dsDNA system (Promega, Madison, WI, USA). A minimum of ~10 ng of DNA was obtained.

Equal amounts of precipitated DNA and input samples were used for the qPCR analysis. Quantitative PCR was performed using 0.4 ng of immunoprecipitated or input DNA and 6 biological replicates. Normalized expression values were calculated with the CFX Manager program using a region located far from the promoter as a reference gene, and we used a region in Gapdh for H3K4me3-ChIP normalization. The primers used in this study are listed in Table S4. The enrichment of each target in the precipitated DNA was evaluated by calculating the ratio between the average of the normalized ChIP DNA copies and the average of the normalized DNA copies in the inputs.

### 4.9. Statistical Analyses

We used the minimum number of animals according to the requirements of the EU Ethics Committee. To assess the statistical significance of epithelial hyperplasia lesions in the prostates of the mice, we performed a Chi-square test. Given that the number of biological replicates in each experiment was relatively low, we performed a nonparametric Wilcoxon–Mann–Whitney test to assess the statistical significance of the results of the qPCR experiments, immunofluorescence, and Western blot quantification.

### 4.10. DNA Methylation Analysis

We analyzed the epigenetic state of genes using our previously published data, GSE235215 [17]. To this end, the normalized counts of reads of methylation peaks located near the promoter of the genes were compared, log transformed, and plotted using the pheatmap library version 1.0.13 using R version 4.2.1.

## 5. Conclusions

Our study revealed that exposure to thiacloprid induces proliferation and is associated with epigenetic alterations in the sperm of genes important for prostate development. Notably, the analysis of markers (Ki-67, PHH3, and HDAC1) showed the effects in F1 but not in F3. However, there are some alterations in *Hox* genes (gene expression, histone H3K4me3 occupancy, sperm DNA methylation level) and in some other factors, such as HDAC1, suggesting that certain alterations could be persistent and possibly impact the prostate at a later age in F3 animals.

## 6. Limitations of This Study

We analyzed murine prostates in young 2-month-old animals. We had to reduce the number of animals used in our studies to follow the requirements of the EU Ethics Committee; thus, the prostates in this study were recovered from other “testis” studies. Prostate pathologies normally appear at a later age; thus, we suggest that some alterations could be observable at a later age, but we did not study them.

## Figures and Tables

**Table 1:** Quantitative analysis of the prostate.

F1	Control	*Thia*	Total	*p*-Value, Chi-Square
Normal	16	19	35	
EPH	1	7	8	0.08
Total	17	26	43	
**F3**	**Control**	***thia***	**Total**	***p*-Value**
Normal	13	20	33	
EPH	1	3	4	0.575
Total	14	23	37	

## Data Availability

The original contributions presented in this study are included in the article/Supplementary Material. Further inquiries can be directed to the corresponding author.

## Supplementary Materials

The following supporting information can be downloaded at https://www.mdpi.com/article/10.3390/ijms27072921/s1.
