# Supplementary material for: Intergenerational Effects of Neonicotinoid Thiacloprid in Murine Prostate Tissue Are Associated with Epigenetic Alterations in Homeobox Hox Genes
Source: Int J Mol Sci. 2026 Mar 24;27(7):2921. doi: 10.3390/ijms27072921 (PMC13072867; doi:10.3390/ijms27072921)
Supplement: Supplementary file 1 [file ijms-27-02921-s001.zip › ijms-4177263-supplementary.pdf]

Intergenerational Effects of Neonicotinoid Thiacloprid in Murine Prostate Tissue Are Associated with Epigenetic Alterations in Homeobox Hox Genes

Ouzna Dali [1,†], Shereen Cynthia D'Cruz [1,†], Chaima Diba Lahmidi [1], Tayeb Mohammed Belkhir [1], Theo De Gestas [1], Christine Kervarrec [1], Pierre-Yves Kernanec [1] and Fatima Smagulova [1,2,*]

1 Université de Rennes, EHESP, Inserm, Irset (Institut de Recherche en Santé, Environnement et Travail), UMR_S 1085, F-35000 Rennes, France
2 Université de Rennes, OSS, Inserm, UMR_S 1242, 35000 Rennes, France
* Correspondence: fatima.smagulova@inserm.fr
† These authors contributed equally to this work.

Supplementary information

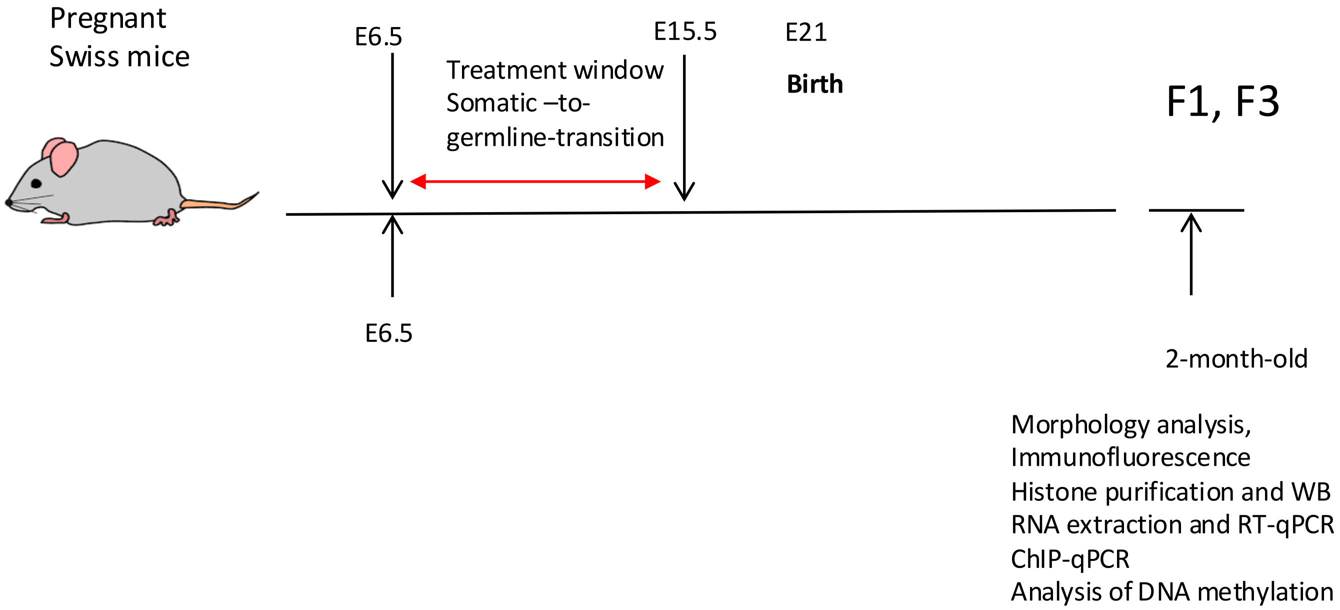

Figure S1. Schematic representation of the experiments. Pregnant outbred Swiss mice were treated from E6.5 to E15.5, with doses of 6 mg/kg/day; control mice received only vehicle (oil). F1 and F3 mice were sacrificed at the age of 2 months. The schema of breeding is described in the "Mice treatment and dissection" section of the Materials and Methods.

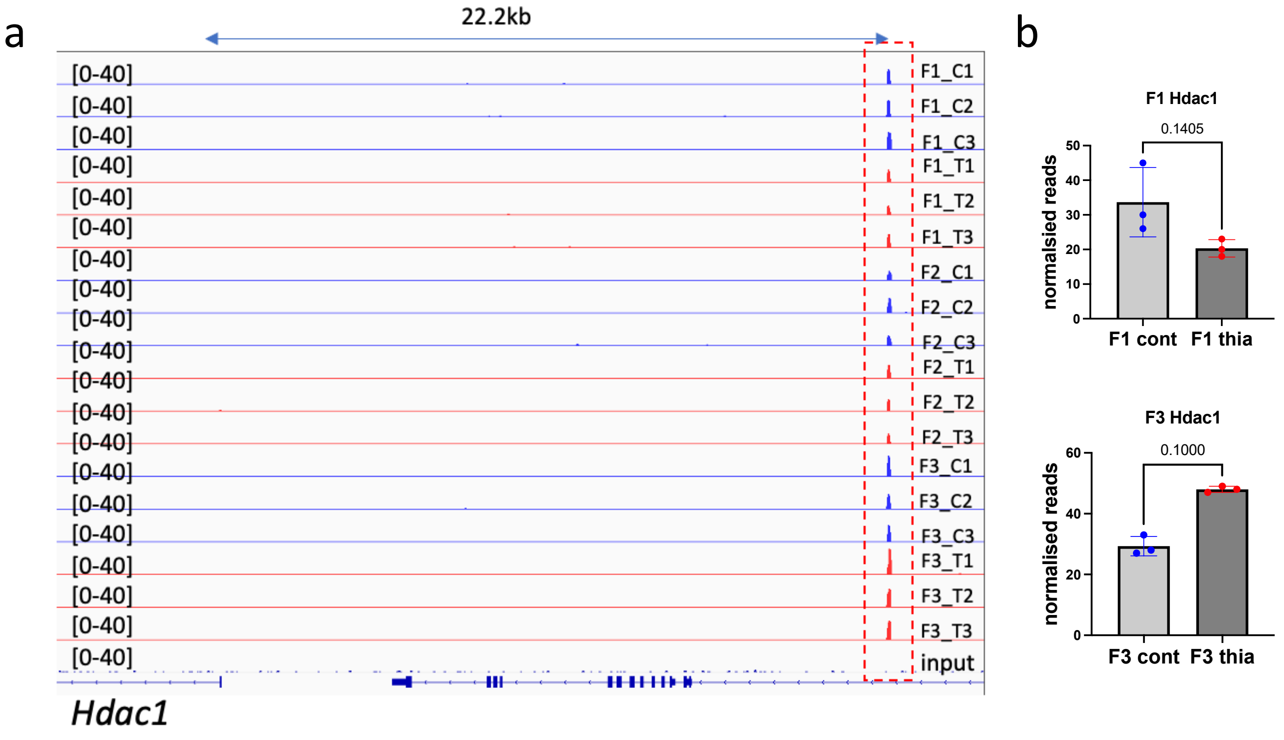

Figure S2. DNA methylation analysis of the *Hdac1* gene in the sperm of F1, F2, and F3 mice. (a) Plots of sequencing reads near the Hdac1 gene; the signal range is indicated in brackets. Normalized counts were averaged and plotted, f1c1-f1c3 are F1 controls, f1t1-f1t3 are F1 treatment samples, f2c1-f2c3 are F2 controls, f2t1-f2t3 are F1 treatment samples, 3c1-fc3 are F3 controls, and f3t1-f3t3 are F3 treatment samples. The sequencing analysis was performed using sperm DNA, with a minimum of 3 replicates for each group. The differential peak is marked by the red dashed box. (b) Quantitative analysis of *Hdac1* counts in F1 (top) and F3 (bottom) graphs. The exact p-value is indicated at the top of the graph; Mann–Whitney test.

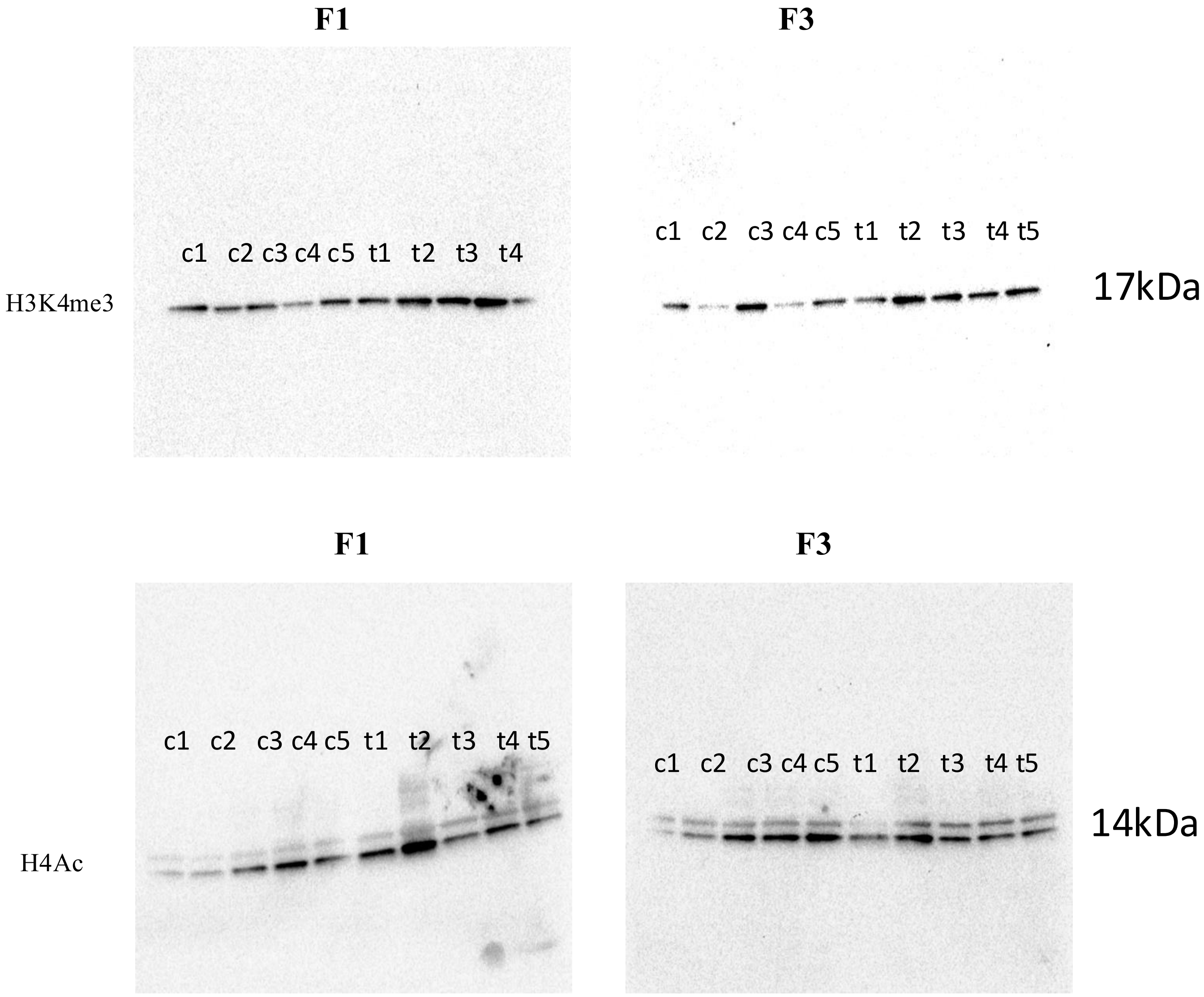

Figure S3. Uncut WB images

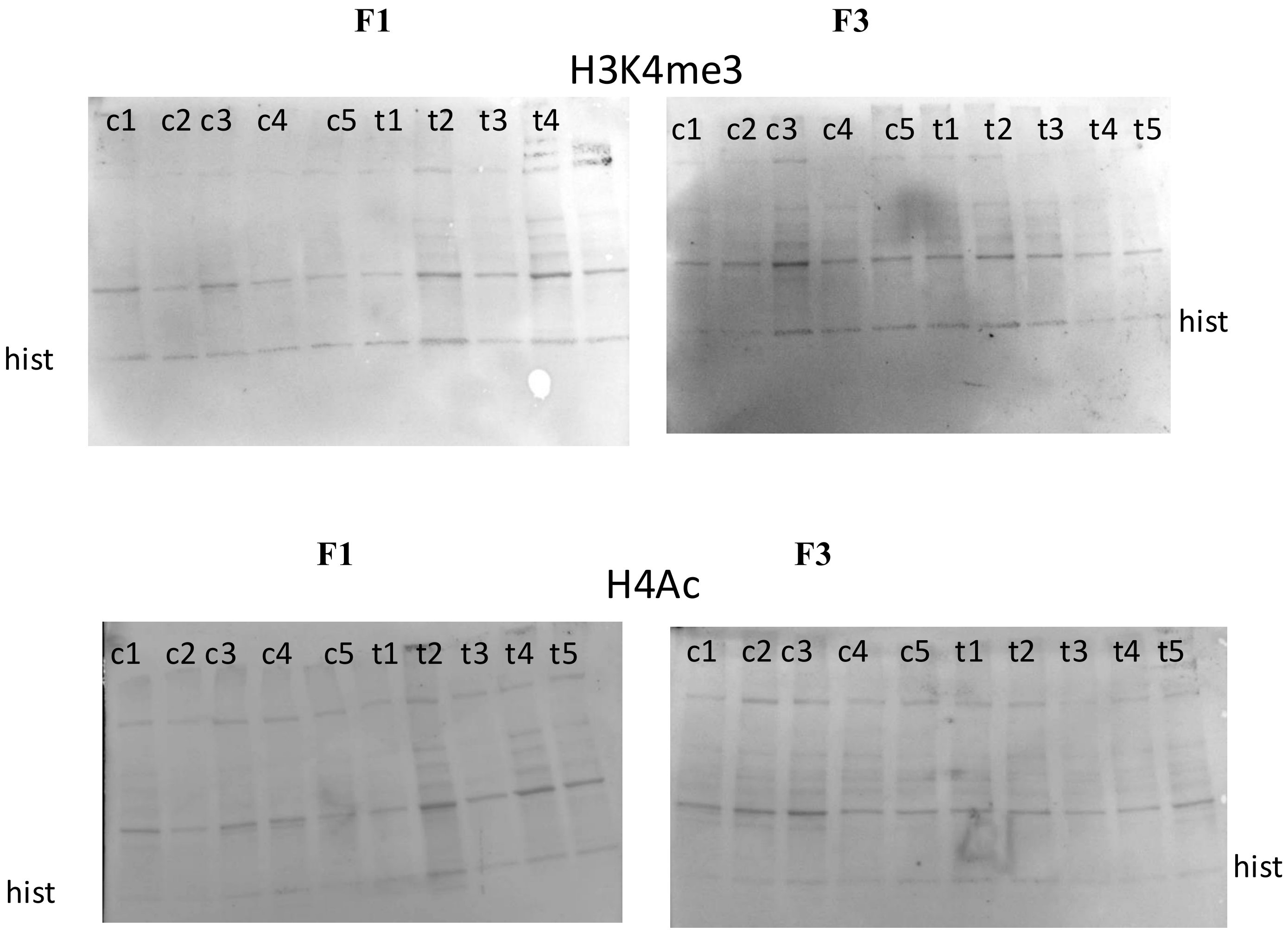

Figure S4. Ponceau red -stained membrane images

Table S1. Biological process “prostate gland development”

Ahr	aryl-hydrocarbon receptor
Apc	APC, WNT signaling pathway regulator
Ar	androgen receptor
Bmp4	bone morphogenetic protein 4
Cd44	CD44 antigen
Cdkn1b	cyclin dependent kinase inhibitor 1B
Ctnnb1	catenin beta 1
Cyp19a1	cytochrome P450, family 19, subfamily a, polypeptide 1
Cyp7b1	cytochrome P450, family 7, subfamily b, polypeptide 1
Eaf2	ELL associated factor 2
Esr1	estrogen receptor 1 (alpha)
Esr2	estrogen receptor 2 (beta)
Fem1b	fem 1 homolog b
Fgf10	fibroblast growth factor 10
Fgfr2	fibroblast growth factor receptor 2
Fkbp4	FK506 binding protein 4
Foxa1	forkhead box A1
Frs2	fibroblast growth factor receptor substrate 2
Gli2	GLI-Kruppel family member GLI2
Hoxa7	zhomeobox A7
Hoxa9	homeobox A9
Hoxa10	homeobox A10
Hoxa13	homeobox A13
Hoxb13	homeobox B13
Hoxd13	homeobox D13
Id4	inhibitor of DNA binding 4
Igf1	insulin-like growth factor 1
Igf1r	insulin-like growth factor I receptor
Mmp2	matrix metallopeptidase 2
Nkx3-1	NK3 homeobox 1
Nog	noggin
Notch1	notch 1
Plag1	pleiomorphic adenoma gene 1
Plaur	plasminogen activator, urokinase receptor
Prlr	prolactin receptor
Psap	prosaposin
Psapl1	prosaposin-like 1
Pten	phosphatase and tensin homolog
Rarg	retinoic acid receptor, gamma
Rln1	relaxin 1
Rxra	retinoid X receptor alpha
Serpinb5	serine (or cysteine) peptidase inhibitor, clade B, member 5
Serpinf1	serine (or cysteine) peptidase inhibitor, clade F, member 1
Sfrp1	secreted frizzled-related protein 1
Shh	sonic hedgehog
Sox9	SRY (sex determining region Y)-box 9
Stat5a	signal transducer and activator of transcription 5A
Stk11	serine/threonine kinase 11
Tnc	tenascin C
Trp63	transformation related protein 63
Ube3a	ubiquitin protein ligase E3A
Wdr77	WD repeat domain 77

Table S2. Localization of proteins encoded by analyzed genes in the prostate

Fgfr2	Basal prostatic cells
Srd5a1	Basal prostatic cells
Notch1	Lymphatic endothelial cells
Ezh2	macrophage
Ctnnb1	Monocytes
Chd1	Neutrophils
Kmt2d	Neutrophils
Ep300	Neutrophils
Stat5a	Neutrophils
Kat2b	NK-cells
Esr1	Pericytes
Hoxa9	prostatic club cells
Sox9	prostatic club cells
Kiss1	prostatic club cells
Nkx3-1	prostatic glandular cells
Foxa1	prostatic glandular cells
Ar	prostatic glandular cells
Hoxb13	prostatic glandular cells
Ahr	prostatic hillock cells
Brca1	ubiquitous
Hdac1	ubiquitous
Ccnd1	ubiquitous
Hoxa7	vascular smooth muscle cell
Hoxa10	vascular smooth muscle cell

Table S3. Oligonucleotides used for RT-qPCR

gene	forward	reverse	biological process
Ahr	AATCCCACATCCGCATGATTAAGAC	TGAGTGGCGATGATGTAATCTGGT	prostate gland development
Akr1b3	CAAACCTTCATCCACTAGTTGTTCC	GGCCCGACTATTTCCCACTG	enables aldose reductase (NADPH) activity
Ar	GTCCTTCACTAATGTCAACTCCA	CCACTGGAATAATGCTGAAGAG	activation of prostate induction by androgen receptor signaling
Brca1	CCCAAAGAAGTAATGACCGTG	GCTAACTATCCACTTTCCTCCTG	regulation of DNA damage checkpoint
Ccnd1	GGACGTCGTCAGGAGCAC	ACCGACGTGCGAGATGTG	G1/S transition of mitotic cell cycle
Chd1	TCATAAACCAACACAGTAATTGCC	GTTGGGATAATAGACCTTGCGT	chromatin organization
Ctnnb1	TCAGTGCAGGAGGCCGAGG	TCCAACTCCATCAGGTCAGCTTG	Wnt signalling
Ep300	CCTTCCACTCCGCTTTCTCA	ACCTTTAGCCTCCTTTGTATCCTC	animal organ morphogenesis
Esr1	CACGTTTCTGTCCAGCACCTTGAAGT	AGAGATGCTCCATGCCTTTGTTACTCA	estrogen receptor signaling pathway
Ezh2	CAAAGGATACAGACAGTGACAGAG	CCGAGAATTTGCTTCAGAGGAG	chromatin organization
Fgfr2	CTGCCGCCAACACTGTCAAG	TGACGGGACCACACTTTCCA	animal organ morphogenesis
Foxa1	CACCTTGGTAGTAGGCTGGC	TCCTTATGGCGCTACCTTGC	mes.-epi. cell signaling involved in prostate gland development
Hdac1	AGCCATCTTTAAGCCAGTCATGTC	GAAACTCTTCACGAACTCCACAC	chromatin remodeling
Hdac6	CACCGCATTCAGAGGGTTCT	CCTTAAGGTGGGGCCAGAAG	negative regulation of protein acetylation
Hoxa10	CTCGCTAGTCCCTTTCCTGC	TCTAGGACTCGCTCCTTCC	prostate gland development
HoxA7	GCCTCTGAGGAACCCAGTAGA	CTGGGCCCATAGGTAGTTTGG	multicellular organism development
Hoxa9	AGTTCTCTCCTTGGCGGTTG	AGTCAGACTGGAAAGCCAGC	prostate gland development
Hoxb13	GCCAGATGTGTTGCCAAGGT	GCACAGCCGTCGGGAG	epithelial cell maturation involved in prostate gland development
Kat2b	GAGTACCTCTTCACCTGCGT	TGTTCACACCCTGTTCAATACTG	protein acetylation
Kiss1	CGGACCCCAGGAACTCGTTA	GGCATGGCGACGACCTAC	G protein-coupled receptor signaling pathway
Kmt2d	CATCCCTGTCTTCCCAGATACCA	CACCTCACTTCCCTTGCCCT	response to estrogen
Nkx3-1	GACCCACCAAGTATCCGGC	CACTTGCTAAGTCCCCTGGATT	prostate gland development
Notch1	CGACAACCGGCAATGTGTGC	CCACCGGCTCACTCTTCACG	prostate gland epithelium morphogenesis
Sox9	AGGCTGTAAAATGCCACTC	CGCTCCGCCTCCTCCACGAA	male gonad development,
Srd5a1	AGGTACCACTGATGATGCTGC	GAGTGGTGTGGCTTTGCACT	androgen biosynthetic process
Stat5a	CACGTGGAAGAACTTTTACGCC	AGCATGGAGTCCAGCGTTC	prostate gland epithelium morphogenesis

Table S4. Oligonucleotides used for ChIP-qPCR

Akr1b3	GTTACAAACCCGGACCGGCA	GATCCGTCTCTCAGCCGTGC	enables aldose reductase (NADPH) activity
Brca1	TAAAATTCCCGCGCTCTCCG	CGCTGACGTGTCTGGATCTT	regulation of DNA damage checkpoint
Ccnd1	GGCGGATGGTCTCCACTTC	TCCGGAGACCGGCAGTACA	G1/S transition of mitotic cell cycle
Ctnnb1	CCCAGTAGAGGCCACAGTGC	GACCCAGCAAGGTACAGCCC	Wnt signalling
Ep300	CTCGTGGGATCAGTGTTGCT	GGGATGCGGACTCAACAGAA	animal organ morphogenesis
Esr1	GTTCAACTACCCCGAGGGCG	CCCAGGCTGTTGGCACTGAA	estrogen receptor signaling pathway
Ezh2	TGGTAACGGTCTTAACCGCC	GGTCACACGCCTTCCTTTCA	chromatin organization
Fgfr2	AGTGAGATTCCATCTTCCTCTGGA	CCTCTTCAGCGAAGCGGTTA	animal organ morphogenesis
Foxa1	CCAGCTGATCGGAACCATTCT	GTCACTCCCCGTGGAAAACC	mes.-epi. cell signaling inv. in prostate gland development
gene	forward	reverse	biological process
Hdac1	GTAAGATGCTCGCGCTGGCT	CATCCCCTCCCCCACTCCAT	chromatin remodeling
Hdac6	CCTCTAATCTGCGCCCGGAAC	GGCGGACTAGAAAGGTGGGTG	negative regulation of protein acetylation
Hoxa10	GCTTCATTACGCTTGCTGCC	CGGTGGAGGTGGCTACTACG	prostate gland development
Hoxa7	GAACCAGGTTTGGGGACCTC	AAGGGAATGAACACCTACGGC	multicellular organism development
Hoxa9	CCGAGAGCGGTTCAGGTTT	CAGGTATATGCGCTCCTGGC	prostate gland development
Hoxb13	TGTGTGCTTTGAGGGAGCCG	GCGCAAGATCAAGCGCAGAC	epithelial cell maturation involved in prostate gland development
Isl1	AGGTGTGCAGCTGCTTCTCG	GAGTCCCTTTGCCCCGTAGC	involved_in cell differentiation
Kiss1	CGGACCCCAGGAACTCGTTA	GGCATGGCGACGACCTAC	G protein-coupled receptor signaling pathway
Mycn	GCGTTGACGCTCCAGGATGT	CCACGCCCCCTCAAAAGTGT	positive regulation of cell population proliferation
Nfix	GTCTGGGTGCTTGCGGTCTT	GTAGAGTAGCGCGCCTGTGG	involved_in DNA replication
Nkx3-1	CTGCCTGGATCCCGACGTA	AGCTAGCGTCGTAGGGAGTG	prostate gland development
Notch1	TGGTCTCACAGGAGCACCCA	CTTGCCGGGATGGCCTCAAT	prostate gland epithelium morphogenesis
Nr2f2	TGCATGCACTGACTGCCGAA	AGTTTGCCCTGACCAACGGG	nervous system development;
Plaur	CCCCGCAGTGAGCGGATAAG	CATCTCGCTGGAGGCTCTGC	epithelial cell differentiation involved in prostate gland
Srd5a1	ACCCTCCAGGTAGACTAGCG	ACTTGGACATCCGAGCATGG	androgen biosynthetic process
Stat5a	GGTCATCGATGGAACATGGCTA	TAGGGGATTTGTCATTGTGTGGAT	prostate gland epithelium morphogenesis
Zfp36	TCCGTGGTCGGATGACAGGT	TGCTTTACAACAGGGGCGGG	negative regulation of cell differentiation
Zzz3	TTGCCTGGCTGGGAAATCGT	AAGGCCTCACCACAGCACAC	involved_in chromatin organization
Sineb1	AGC CGG GCG TGG TGG	CTTTGT AGA CCA GGC TGG CCT C	retroelement
IAPEz	CTTTGT AGA CCA GGC TGG CCT C	TGCACATAAAGCTGGCACA	retroelement
L1-gf	CTCCTTGGCTCCGGGACT	CAGGAAGGTGGCCGGTTGT	retroelement
l1-T	CAGCGGTCGCCATCTTG	CACCCTCTCACCTGTTCAGACTAA	retroelement
major sat	GACGACTTGAAAAATGACGAAATC	CATATTCCAGGTCCTTCAGTGTGC	satellite DNA
